# The interplay of *Pseudomonas aeruginosa* and *Staphylococcus aureus* in dual-species biofilms impacts development, antibiotic resistance and virulence of biofilms in *in vitro* wound infection models

**DOI:** 10.1371/journal.pone.0304491

**Published:** 2024-05-28

**Authors:** Pia Katharina Vestweber, Jana Wächter, Viktoria Planz, Nathalie Jung, Maike Windbergs

**Affiliations:** Institute of Pharmaceutical Technology, Goethe University Frankfurt, Frankfurt am Main, Germany; University of Pennsylvania, UNITED STATES

## Abstract

Due to high tolerance to antibiotics and pronounced virulence, bacterial biofilms are considered a key factor and major clinical challenge in persistent wound infections. They are typically composed of multiple species, whose interactions determine the biofilm’s structural development, functional properties and thus the progression of wound infections. However, most attempts to study bacterial biofilms *in vitro* solely rely on mono-species populations, since cultivating multi-species biofilms, especially for prolonged periods of time, poses significant challenges. To address this, the present study examined the influence of bacterial composition on structural biofilm development, morphology and spatial organization, as well as antibiotic tolerance and virulence on human skin cells in the context of persistent wound infections. By creating a wound-mimetic microenvironment, the successful cultivation of dual-species biofilms of two of the most prevalent wound pathogens, *Pseudomonas aeruginosa* and *Staphylococcus aureus*, was realized over a period of 72 h. Combining quantitative analysis with electron microscopy and label-free imaging enabled a comprehensive evaluation of the dynamics of biofilm formation and matrix secretion, revealing a twofold increased maturation of dual-species biofilms. Antibiotic tolerance was comparable for both mono-species cultures, however, dual-species communities showed a 50% increase in tolerance, mediated by a significantly reduced penetration of the applied antibiotic into the biofilm matrix. Further synergistic effects were observed, where dual-species biofilms exacerbated wound healing beyond the effects observed from either *Pseudomonas* or *Staphylococcus*. Consequently, predicting biofilm development, antimicrobial tolerance and virulence for multi-species biofilms based solely on the results from mono-species biofilms is unreliable. This study underscores the substantial impact of a multi-species composition on biofilm functional properties and emphasizes the need to tailor future studies reflecting the bacterial composition of the respective *in vivo* situation, leading to a more comprehensive understanding of microbial communities in the context of basic microbiology and the development of effective treatments.

## Introduction

Persistent wound infections *in vivo* are predominantly associated with the presence of bacterial biofilms [1, 2], which are defined as microbial aggregates embedded in a self-produced, hydrated matrix [3, 4]. Wound-related biofilms are characterized by a heterogeneous poly-microbial population, in which the opportunistic pathogens *Pseudomonas aeruginosa* and *Staphylococcus aureus* are particularly widespread with reported prevalence rates of 52.2% and 93.5%, respectively [5]. While those bacteria also occur in acute wound infections as planktonic organisms, the development of persistent infections coincides with the transition to the biofilm phenotype [6]. This complex and dynamic process is initiated by reversible attachment of individual bacteria to the wounded tissue or to each other [7, 8]. Subsequently, bacteria multiply and form bacterial aggregates that strongly adhere to the wound bed [7]. This attachment is mediated by the production of the biofilm matrix which consists of extracellular polymeric substances (EPS), primarily including polysaccharides, proteins, extracellular DNA, and lipids [9, 10]. The collection of these biomolecules, along with their diverse structural and functional attributes, is referred to as the ’matrixome’ [11]. As the bacterial communities grow, the maturation process continues, resulting in larger bacterial aggregates and microcolonies with an intricate and highly organized three-dimensional structure, characterized by high cell density and microbial diversity [4, 7]. As the last step of the biofilm life cycle, motile biofilm bacteria can be dispersed to spread the infection to other areas of the human body [12]. In general, the biofilm development process can take up to several days [13, 14]. However, it is noteworthy that the dynamics of biofilm development are highly dependent on the surface properties and external conditions as well as the bacterial species involved [15–17].

Starting from the stage of irreversible attachment, the structural changes promote the development of functional biofilm properties, that distinguish biofilms from their planktonic counterpart: Especially a pronounced tolerance towards antimicrobial treatments and the host immune response, as well as enhanced virulence including immunomodulatory activity of biofilms can be observed [18, 19]. Mechanisms mediating the tolerance of the biofilm phenotype encompass the matrix, which acts as a physical barrier that impedes antibiotic penetration, the emergence of persister cells with reduced metabolic activity, and biofilm quorum sensing (QS) pathways, as part of a bacteria’s cell-cell communication system that enables the exchange of resistance mechanisms in a mono- or multi-species community [20]. The virulence of biofilms, referring to the degree of pathogenicity, is driven by the matrixome next to multiple exoproducts like toxins and QS molecules [21]. In the context of persistent wound infections, these secreted molecules exert negative effects on host cell viability, proliferation, and migration, as well as the induction of a hyper-inflammatory infection state, directly impeding physiological wound healing [8]. Due to their critical role in the severity of persistent wound infections, biofilms became a focal point in clinical microbiology and related fields of research.

There is growing evidence that bacterial interactions within multi-species biofilms determine the structural development and functional properties of biofilms and thus the progression of wound infections [22–24]. For instance, studies have investigated the impact of co-cultivation *of Pseudomonas aeruginosa* and *Staphylococcus aureus* on their tolerance towards antimicrobial treatments [25–28]. Unfortunately, the results are inconsistent and comparing them retrospectively is complicated by the distinct heterogeneity of the existing literature in terms of bacterial strains, external growth conditions, and cultivation periods. In general, cultivating multiple species within one biofilm represents a challenging task in the *in vitro* environment, especially over extended periods of time [1, 29]. As a result, studies with multi-species biofilms are scarce and mostly employ short cultivation times without consideration of the developmental stage of the biofilms and their related functional properties. The inability of a prolonged co-cultivation of multiple bacterial species over several days might be explained by the use of inappropriate culture substrate, as studies often rely on an artificial growth environment. This issue could be addressed by implementing more sophisticated approaches that accurately replicate the complex *in vivo* microenvironment of infected tissue, which are already widely accepted for the investigation of mono-species biofilms [30–33].

For both, basic microbiology research and the development of effective treatments, considering potential alterations due to a multi-species composition of biofilms is crucial. Remarkably, no single study to date has comprehensibly evaluated the impact of bacterial composition on biofilm development and their functional properties in the context of wound infections within a bio-relevant *in vitro* growth environment. The distinct heterogeneity of the existing literature, due to variations in bacterial strains, microbial diversity, external growth conditions, and cultivation periods, complicates their retrospective comparison. To bridge this gap, we cultivated mono-species biofilms of *P*. *aeruginosa* and *S*. *aureus*, as well as dual-species biofilms of both pathogens over 72 h. To allow the prolonged co-cultivation of both strains, we employed a model system, which was previously developed by our group and closely simulates the microenvironment of wound infections [33]. Particularly, the influence of bacterial composition on structural biofilm development including the morphology and spatial organization as well as antibiotic tolerance and virulence in the context of persistent wound infections were evaluated. This study aimed to understand differences in morphology, composition and growth dynamics between mono species and dual species biofilms as well as their respective antibiotic susceptibility and virulence on human skin cells. By assessing whether findings from mono-species biofilm studies can be extrapolated to multi-species biofilms, and if this predictability is species-dependent, this study offers great potential for advancing basic research as well as for improving therapeutic strategies for persistent wound infections.

## Results

### Investigation of biofilm morphology by scanning electron microscopy

**Fig 1:**
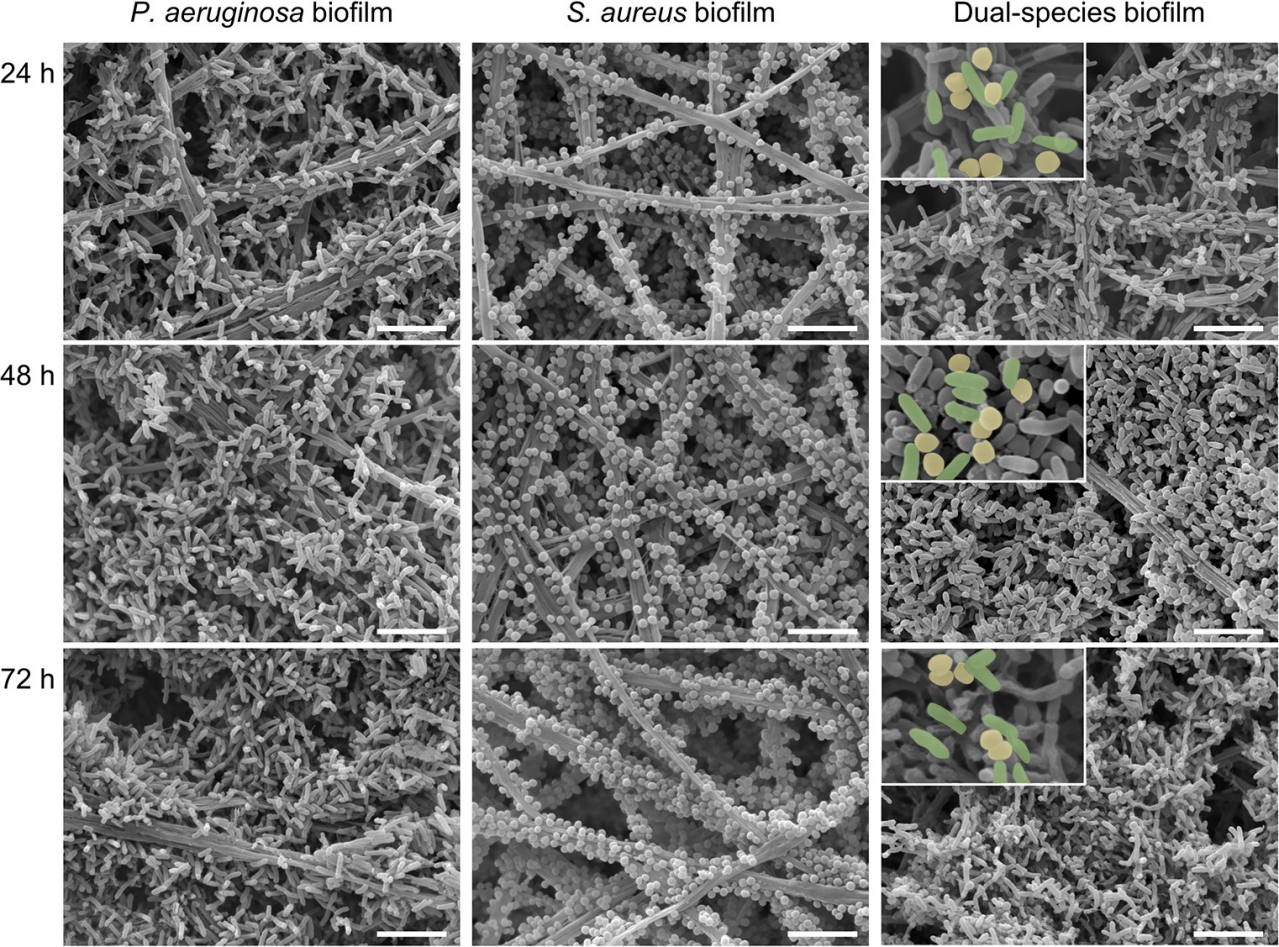
Scanning electron micrographs of biofilms for evaluating morphological changes throughout their development. Representative micrographs are shown for biofilms composed of *P*. *aeruginosa* and *S*. *aureus* as mono-species and dual-species biofilms. All biofilms were cultivated for 24, 48, and 72 h prior to evaluation. Scale bar = 5 μm.

In this study, bacterial biofilms were cultivated within a three-dimensional electrospun nanofibrous scaffold as substrate. This model system was previously developed by our group and allows to resemble a microenvironment comparable to that of *in vivo* wound infections in combination with ex vivo human skin [33]. For illustration, S1 Fig shows a representative micrograph of the nanofibrous scaffold, acquired with scanning electron microscopy (SEM). SEM analysis of the biofilms was conducted with mono-species biofilms of *P*. *aeruginosa* and *S*. *aureus* as well as dual-species biofilms after cultivation times of 24, 48, and 72 h to examine biofilm formation and time-dependent changes in biofilm morphology. Representative micrographs are shown in Fig 1. After 24 h, within mono-species biofilms of *P*. *aeruginosa* the rod-shaped bacteria adhered to the scaffold predominantly as single organisms. However, after 48 and 72 h, a compact biofilm structure was visible, consisting of large bacterial aggregates, with no considerable morphological differences observed between these time points. The amount of coccoid-shaped *S*. *aureus* adhering to the fibers within mono-species biofilms increased gradually from 24 to 72 h. While they tended to colonize the substrate as individual bacteria at the first time points, bacterial aggregates mainly formed after 72 h. However, a distinct overall porosity of these biofilms remained, whereby the nanofibrous scaffold stayed visible. Dual-species biofilms were characterized by a compact morphology of high cell density throughout all time points, particularly after 48 and 72 h, completely covering the fibrous scaffolds. Within these biofilms, *P*. *aeruginosa* and *S*. *aureus* were observed to coexist in close proximity (visualized in Fig 1 by different colors). Additionally, in *P*. *aeruginosa* mono-species biofilms as well as dual-species biofilms, thin secondary fiber networks in between the bacteria were observed after 48 h, representing the EPS of biofilm matrix (S2 Fig). In contrast, in *S*. *aureus* mono-species biofilms no visible EPS were evident.

### Analysis of biofilm bacterial growth by quantification of colony forming units

**Fig 2:**
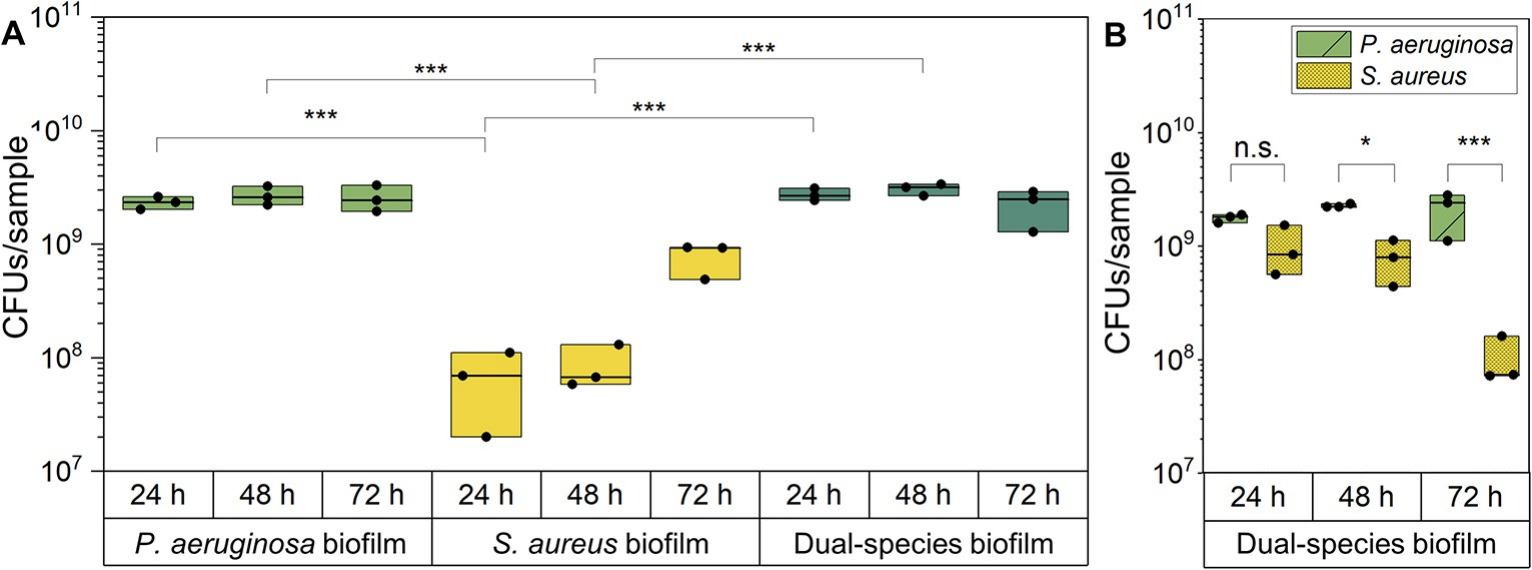
Quantification of colony forming units of adherent biofilm bacteria. (A) Mono-species biofilms of *P*. *aeruginosa* and *S*. *aureus* as well as dual-species biofilms of both species were cultivated for 24, 48 and 72 h. After each time point, the total number of viable, adherent bacteria was determined. (B) For dual-species biofilms, their bacterial composition was evaluated after 24, 48 and 72 h of co-cultivation. *p < 0.05, **p < 0.01, and ***p < 0.001.

In order to quantitatively assess the development of mono-species biofilms of *P*. *aeruginosa* and *S*. *aureus* as well as dual-species biofilms, all model systems were inoculated with an equal total bacterial load adjusted to 1.5*10^6^ cells. Subsequently, colony forming units (CFUs) of adherent bacteria were determined over a cultivation period of 72 h (Fig 2). Total counts of viable bacterial are illustrated in Fig 2A. For *P*. *aeruginosa* mono-species biofilms, high numbers of adherent biofilm bacteria were already apparent after 24 h with about 2.3*10^9^ CFUs / biofilm, not significantly changing over 48 and 72 h, respectively. Conversely, the quantity of adherent *S*. *aureus* in mono-species biofilms was comparably low at 24 h (6.6*10^7^ CFUs / biofilm) and exhibited a gradual increase throughout the remaining cultivation time with a peak at approximately 7.8*10^8^ CFUs / biofilm after 72 h of cultivation. The total bacterial load of dual-species biofilms was comparable to that of *P*. *aeruginosa* mono-species biofilms at all three time points, showing no significant alteration over time with determined CFUs ranging from 2.2*10^9^ to 3.1*10^9^. At all time points, the quantified CFUs of *S*. *aureus* mono-species biofilms were significantly reduced compared to both, *P*. *aeruginosa* biofilms and dual-species biofilms. To gain a more comprehensive understanding of the bacterial composition within dual-species biofilms, the CFUs / biofilm of *P*. *aeruginosa* and *S*. *aureus* were determined by using strain-specific agar plates (Fig 2B). After 24 h of co-cultivation, the number of adherent bacteria did not significantly differ between *S*. *aureus* and *P*. *aeruginosa*, even though slightly more CFUs were determined for *P*. *aeruginosa* reaching 1.8*10^9^ CFUs / biofilm (equals 65% of total bacterial count) compared to 9.7*10^8^ CFUs / biofilm (equals 35% of total bacterial count) for *S*. *aureus*. After 48 and 72 h, *P*. *aeruginosa* increasingly dominated the dual-species biofilms, exhibiting significantly higher numbers of CFUs than *S*. *aureus*. The greatest disparities were observed after 72 h, where *P*. *aeruginosa* accounted for 2.1*10^9^ CFUs / biofilm (equals 95% of total bacterial count) while solely 1.0*10^8^ CFUs / biofilm were determined for *S*. *aureus*. It is noteworthy that after 24 and 48 h, more adherent *S*. *aureus* bacteria were counted in dual-species biofilms compared to mono-species biofilms.

### Imaging of hydrated biofilms combined with molecular analysis by confocal Raman microscopy

**Fig 3:**
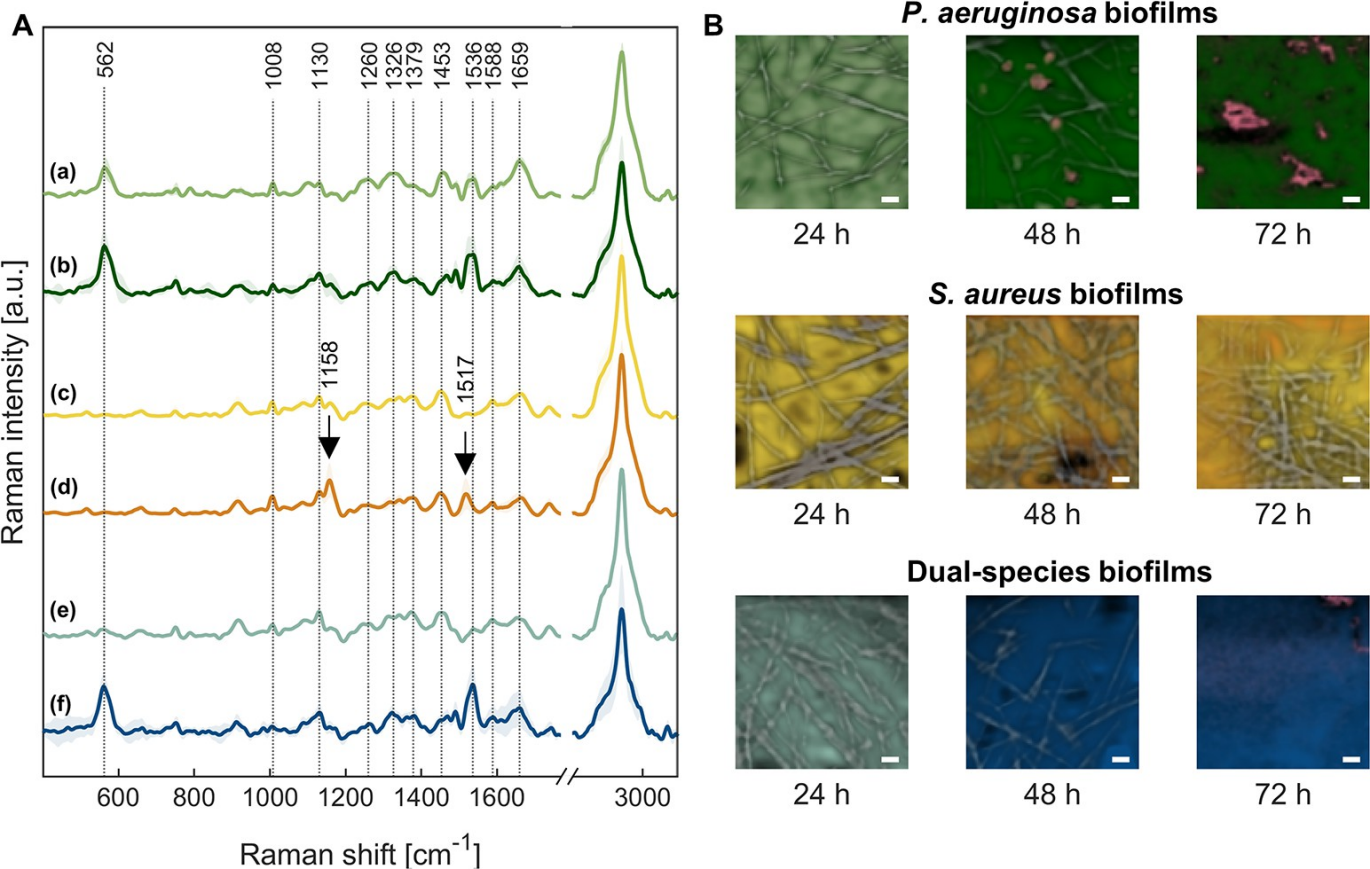
Raman spectra and false color images of different biofilms. Raman scans of the three different biofilms were acquired on their surfaces after 24, 48 and 72 h. (A) Representative Raman peaks are highlighted in the mean biomass spectra of the samples. (B) False color images revealed the distribution of biomass (shown in light and dark green for *P*. *aeruginosa*, yellow and orange for *S*. *aureus* and light and dark blue for the dual-species biofilm) and fluorescent regions (shown in pink) on the fiber network (shown in grey). Scale bar = 5 μm.

To avoid harsh sample preparation as required for SEM analysis and to enable evaluation of biofilms in their hydrated state, confocal Raman microscopy was implemented in this study. Raman scans were acquired on the surface of the three different biofilms after 24, 48 and 72 h and subsequently subjected to variance component analysis, revealing spectral endmembers which were further assigned to the fiber network and the respective biomass consisting of bacteria and extracellular matrix (Fig 3).

Independent of the bacterial composition of the biofilm, the Raman spectrum of the fiber network was characterized by Raman signals of cellulose acetate and gelatin (S3 Fig) [33–36]. Raman scans acquired from *P*. *aeruginosa* biofilms revealed two distinct groups of spectra that were assigned to the biomass. The corresponding mean spectra are depicted in Fig 3Aa and 3Ab, both were characterized by typical Raman peaks of biological bacterial components. Signals at 1008, 1260, 1611 and 1659 cm^-1^ were assigned to phenylalanine (symmetric ring breathing), amide III, tyrosine (C = C stretching) and amide I, respectively, indicating the presence of proteins [37–39]. Peaks detected at 1326 and 1457 cm^-1^ were linked to the C-H deformation of lipids and carbohydrates [37, 39]. Additional signals at 1130 and 1379 cm^-1^ corresponded to glycosidic rings (C-C and C-O-C stretching of glycosidic linkages) and peptidoglycan (symmetric and asymmetric stretching of C-O-O) [37, 39]. Nucleic acid peaks were identified at 1100 (symmetric stretching of PO_2_^-^) and 1588 cm^-1^ (adenine and guanine) [37, 39]. The two spectra primarily differ in the intensity of the peaks at 567 and 1544 cm^-1^, suggesting the presence of polysaccharides (C-O-C glycosidic ring deformation) and pigments such as the phenazine pyocyanin (C = C stretching, ring deformation), respectively. The second spectrum shows significantly higher levels of these compounds (Fig 3Ab) [33, 37, 40, 41]. After 24 h of cultivation, the fiber network remained clearly visible, while the biomass was primarily defined by the first spectrum (Fig 3B). Over the cultivation period, the amount of biomass described by the second spectrum increased and dominated the false color image after 72 h, covering the fibers and completely filling the gaps within the fiber network. In addition to the biomass, strongly fluorescent areas appeared after 48 h (Fig 3B, shown in pink).

Raman scans of *S*. *aureus* biofilms also showed two distinct groups of biomass spectra (Fig 3Ac and 3Ad). Again, the spectra were characterized by the Raman peaks described above for the biological bacterial components. In the second biomass spectrum, two additional peaks were observed at 1158 and 1517 cm^-1^, corresponding to the vibrations of carotenoid pigments (= C-C = and -C = C) [34]. The false color images revealed biomass surrounding the clearly visible fiber network throughout all time points. After 24 h, the biomass spectrum lacking carotenoid contribution was dominant, while carotenoid-containing areas increased after 48 and 72 h.

The two Raman spectra of dual-species biofilms exhibited similar signals as the spectra acquired on *P*. *aeruginosa* biofilms (Fig 3Ae and 3Af). However, the first spectrum lacked the peaks at 567 and 1544 cm^-1^. No carotenoid peaks specific to *S*. *aureus* were present in any of the spectra. Accordingly, no separation of the different species was possible in the false color images (Fig 3B). An increase in biomass was observed with the second spectrum becoming more dominant over time. After 72 h, the fiber network was entirely covered by biomass.

### Assessment of biofilm tolerance by antibiotic susceptibility testing

**Fig 4:**
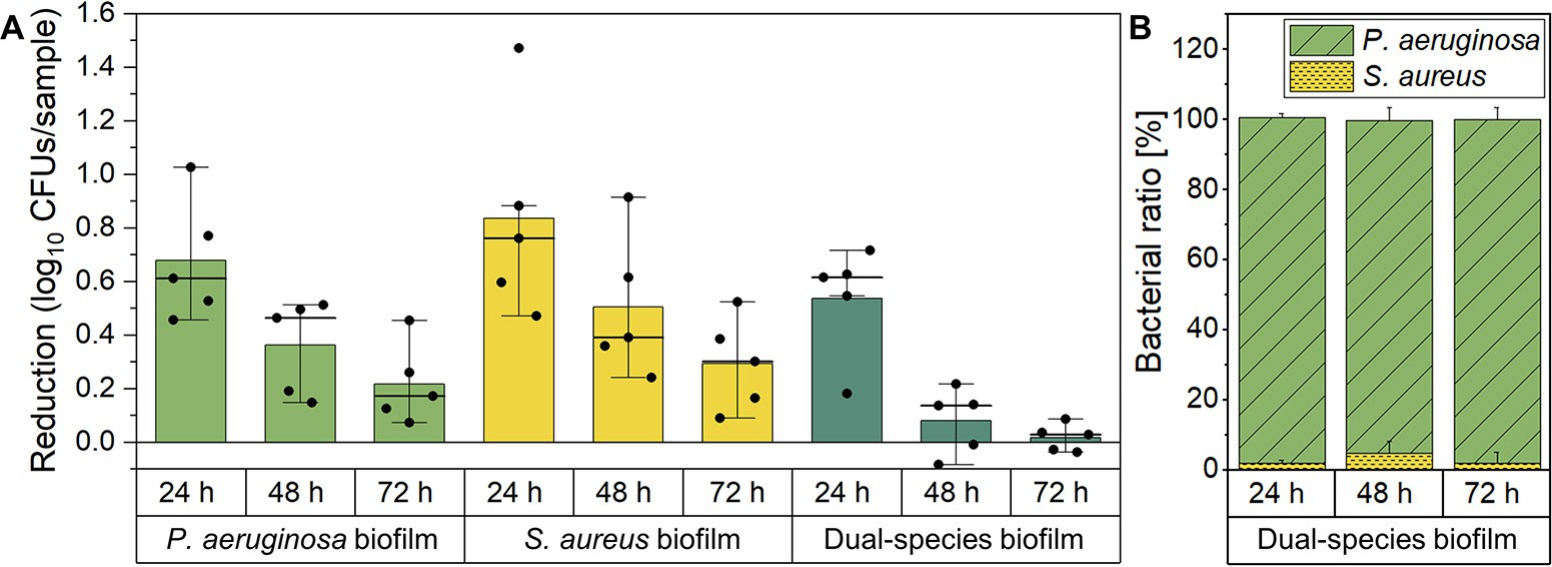
Susceptibility testing of the biofilms to the antibiotic gentamicin. (A) *P*. *aeruginosa* and *S*. *aureus*, cultivated as mono-species and dual-species biofilms for 24, 48 or 72 h, were treated with a 10 μg/mL gentamicin solution for 24 h. Subsequently, CFUs were quantified and compared to the untreated control. (B) For dual-species biofilms, the ratio of both species was determined after the treatment with gentamicin.

To evaluate the impact of bacterial composition on the susceptibility of the different biofilms to an antimicrobial treatment, tolerance testing to a 10 μg/mL gentamicin solution was conducted for a treatment period of 24 h (Fig 4). The antibiotic gentamicin was selected due to its regular application in clinical practice for the treatment of tissue infections. It was applied in a concentration above the minimal inhibition concentration (MIC) for susceptibility testing to ensure antimicrobial efficacy against *P*. *aeruginosa* and *S*. *aureus* (MIC (*P*. *aeruginosa*): 2 μg/mL, MIC (*S*. *aureus*): 0.25 μg/mL) [42]. To further verify the selected concentration in the particular experimental setting, planktonic bacteria of both strains were subjected to the treatment, resulting in a reduction of viable bacteria by more than 1000-fold (S1 File). In case of mono-species biofilms of *P*. *aeruginosa*, the reduction of viable bacteria after antibiotic treatment decreased gradually with the duration of biofilm cultivation, reaching a minimum of approximately 0.2 log_10_ CFUs/sample for biofilms cultivated for 72 h (Fig 4A). A comparable trend was observed for *S*. *aureus* biofilms with a slightly higher reduction of viable bacteria at all time points and a minimal reduction of approximately 0.3 log_10_ CFUs/sample for biofilms cultivated for 72 h. For all cultivation periods, the dual-species biofilms exhibited an improved tolerance compared to the mono-species biofilms. Already for biofilms cultivated for 48 h, a reduction of approximately only 0.08 log_10_ CFUs/sample was observed, which further decreased to 0.02 log_10_ CFUs/sample for 72 h biofilms. To evaluate the antibiotic tolerance of the individual species within the dual-species biofilms, the numbers of viable *P*. *aeruginosa* and *S*. *aureus* were determined by using strain specific agar plates for quantification after the antibiotic treatment. Within the dual-species biofilms, *P*. *aeruginosa* dominated over *S*. *aureus* after gentamicin treatment (Fig 4B). While for biofilms cultivated for 24 and 72 h only 1,94% and 1,87% of viable bacteria accounted for *S*. *aureus*, respectively, its tolerance within dual-species biofilms was slightly improved for biofilms cultivated for 48 h, reaching a viability of 4.89%.

### Investigation of gentamicin penetration into the biofilms by confocal Raman microscopy

**Fig 5:**
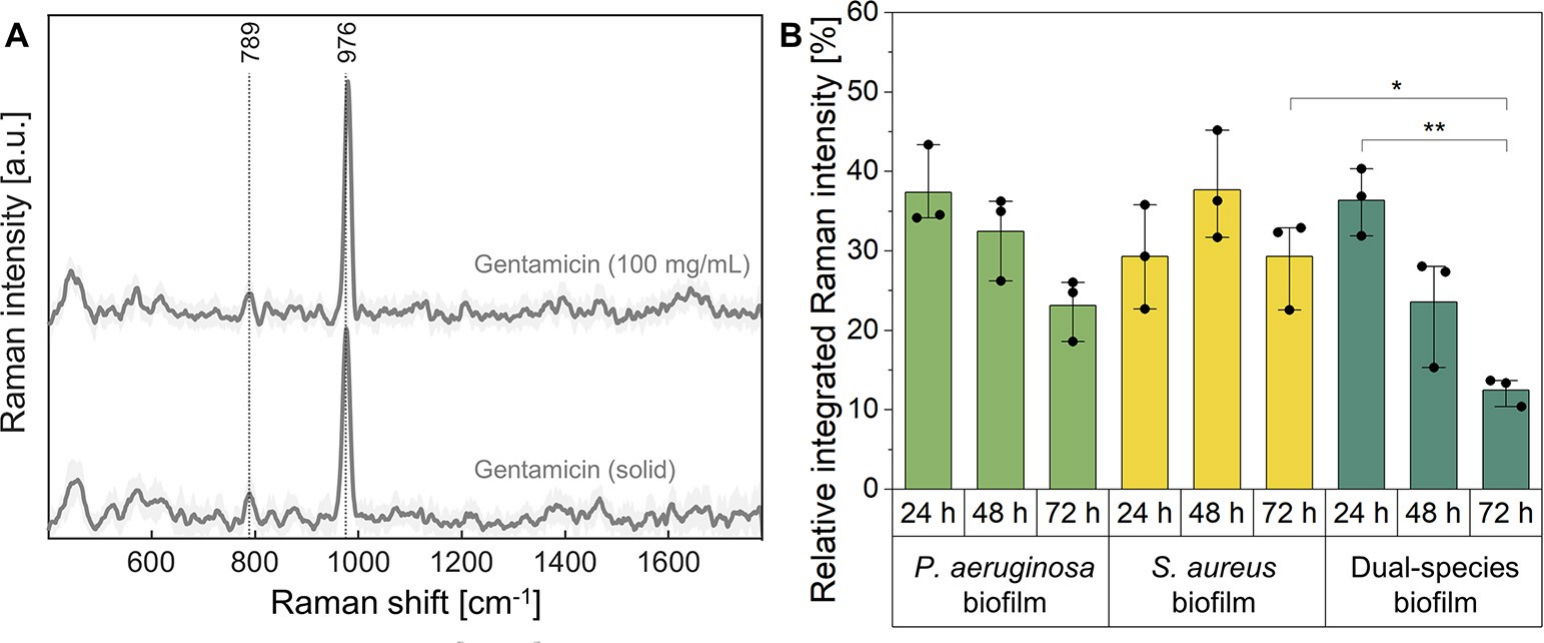
Raman analysis of the penetration ability of gentamicin into the biofilms. Fixed mono- and dual-species biofilms were incubated with a 100 mg/mL gentamicin solution. For every Raman spectrum, the area under the curve (AUC) of a distinct gentamicin peak was determined and the ratio between the gentamicin applied and the AUC of the gentamicin in the biofilm was calculated.

Raman microscopy was used to investigate the gentamicin penetration into the different biofilms. After 24, 48 and 72 h of cultivation, fixed mono- and dual-species biofilms were treated with a 100 mg/mL gentamicin solution. This concentration was used to ensure sufficient signal intensity. For every spectrum, the area under the curve was calculated for the most pronounced Raman signal of gentamicin at wavenumber 974 cm^-1^, corresponding to C-O-C stretching of the active (Fig 5A, a schematic overview of the procedure can be found in S4 Fig) [43]. Fig 5B displays the ratio between the integrated Raman intensity of the gentamicin applied and the antibiotic detected inside the biofilm. In case of the mono-species biofilms of *P*. *aeruginosa*, about 37.4% of the gentamicin applied was overall detected in the 24 h cultivated biofilms. The amount decreased with increasing cultivation time down to 23.1% for the biofilms cultivated for 72 h. Within the *S*. *aureus* biofilms, no significant reduction in penetration was observed, with ratios of 29.3%, 37.7% and 29.3% for 24, 48 and 72 h cultivated biofilms, respectively. The most pronounced impact on penetration was observed for the dual-species biofilms. While the ratios for the 24 and 48 h cultivated biofilms were still comparable to the mono-species biofilms, a significantly reduced gentamicin signal was observed in the biofilms cultivated for 72 h, resulting in a ratio of 12.5%.

### Analysis of biofilm virulence on *in vitro* wound healing assays

**Fig 6:**
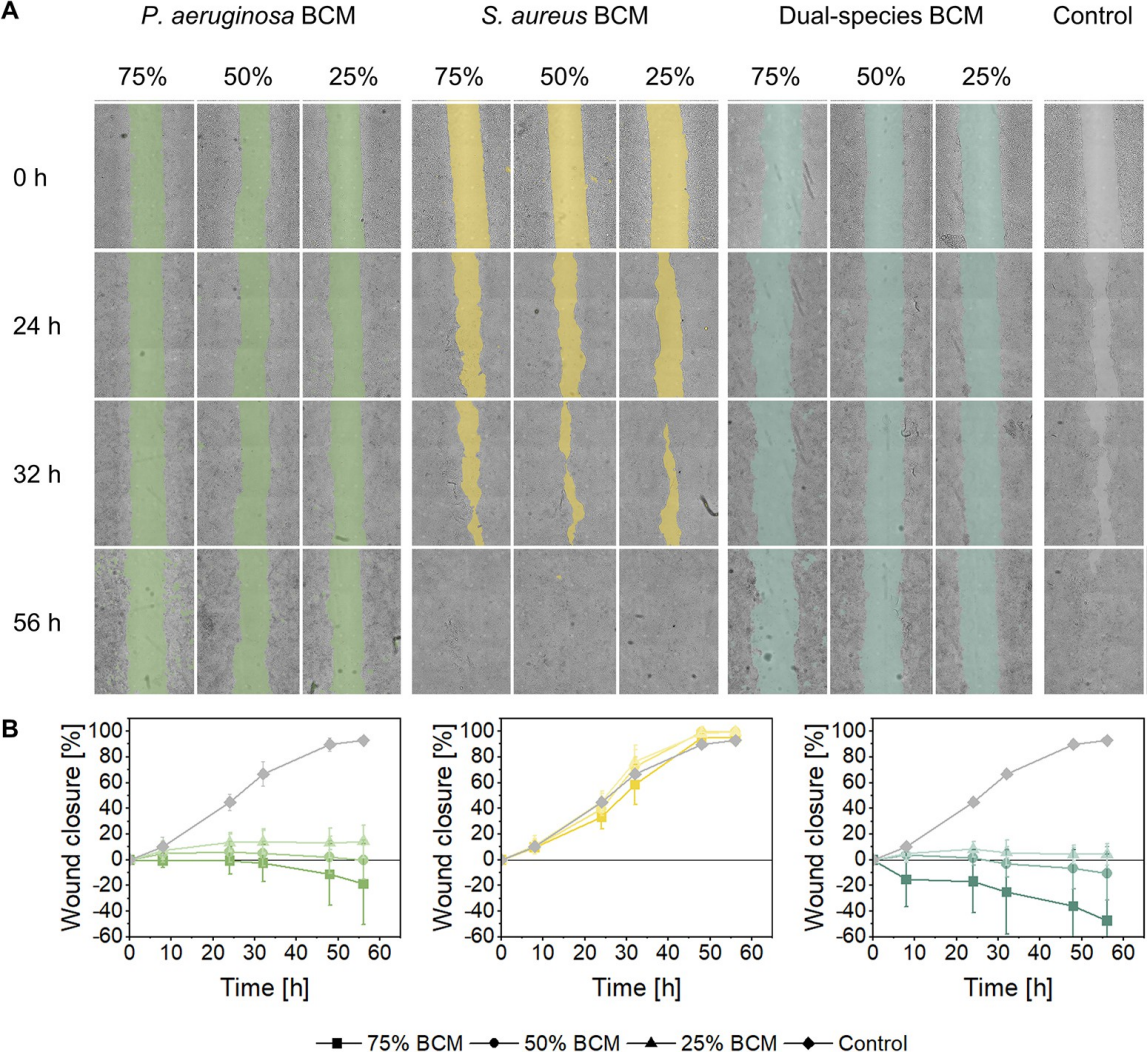
Scratch assays for evaluating the virulence of biofilm components regarding their effect on wound healing. A confluent monolayer of human skin keratinocytes were wounded and then treated with biofilm-conditioned media from mono-species biofilms of *P*. *aeruginosa* and *S*. *aureus* as well as dual-species biofilms. Concentrations of 75%, 50%, and 25% were applied. Further, (A) acquired images, from which representatives are shown, were used for calculating the (B) wound closure relative to the initial wound area.

To assess the virulence of the different biofilms depending on their bacterial composition, their impact on wound healing was investigated using an *in vitro* scratch wound healing assay with human keratinocytes incubated with biofilm conditioned media (BCM) in concentrations from 25% to 75% (Fig 6A). For cells treated with BCM derived from *P*. *aeruginosa* (PA BCM), a notable effect on wound closure was observed across all concentrations (Fig 6B). Already after 8 h, wounds treated with 75% PA BCM exhibited not only an impaired wound healing but a detachment of cells on wound edges and the surrounding cell layer, leading to an increase in wound size, which continued throughout the observation period. Wounds treated with either 25% or 50% PA BCM did not show a significant difference compared to the control cultures after 8 h. However, after 24 h, the impact of these dilutions also became significant, with 6.0% and 13.5% closure of the samples treated with 50% and 25% PA BCM, respectively, while the control cultures showed 44.8% wound closure. During the subsequent observation period, samples exposed to 50% PA BCM showed an increased wound size, resulting in a final wound closure of -0.1% after 56 h. The closure of wounds treated with 25% PA BCM continued up to 14.3%, leading to significant differences compared to the control cultures at all concentrations. For all concentrations applied, cells treated with BCM derived from *S*. *aureus* (SA BCM) displayed a wound healing comparable to the control group (Fig 6B). For 50% and 25% BCM after 32 h and for all concentrations after 48 h wound closure was even slightly accelerated (statistically not significant). After 56 h, the sample as well as the control cultures reached a final wound closure of about 95%.

The medium conditioned by biofilms containing both species (Dual BCM) had the most pronounced impact on wound closure (Fig 6B). 75% of conditioned medium led to an expansion in wound sizes, beginning after 8 h with -15.2%, while scratches treated with 25% or 50% Dual BCM did not show a significant difference compared to the control cultures at this time point. The wound size exposed to 50% Dual BCM increased after 24 h and even exceeded the initial wound area after 48 h. The wound closure when treated with 25% Dual BCM did not exhibit further changes during the subsequent observation period, ranging between 4.3% and 8.2%, resulting in significant differences compared to the control cultures at all concentrations at 56 h.

### Assessment of the pro-inflammatory response and viability of host cells after treatment with biofilm conditioned medium

**Fig 7:**
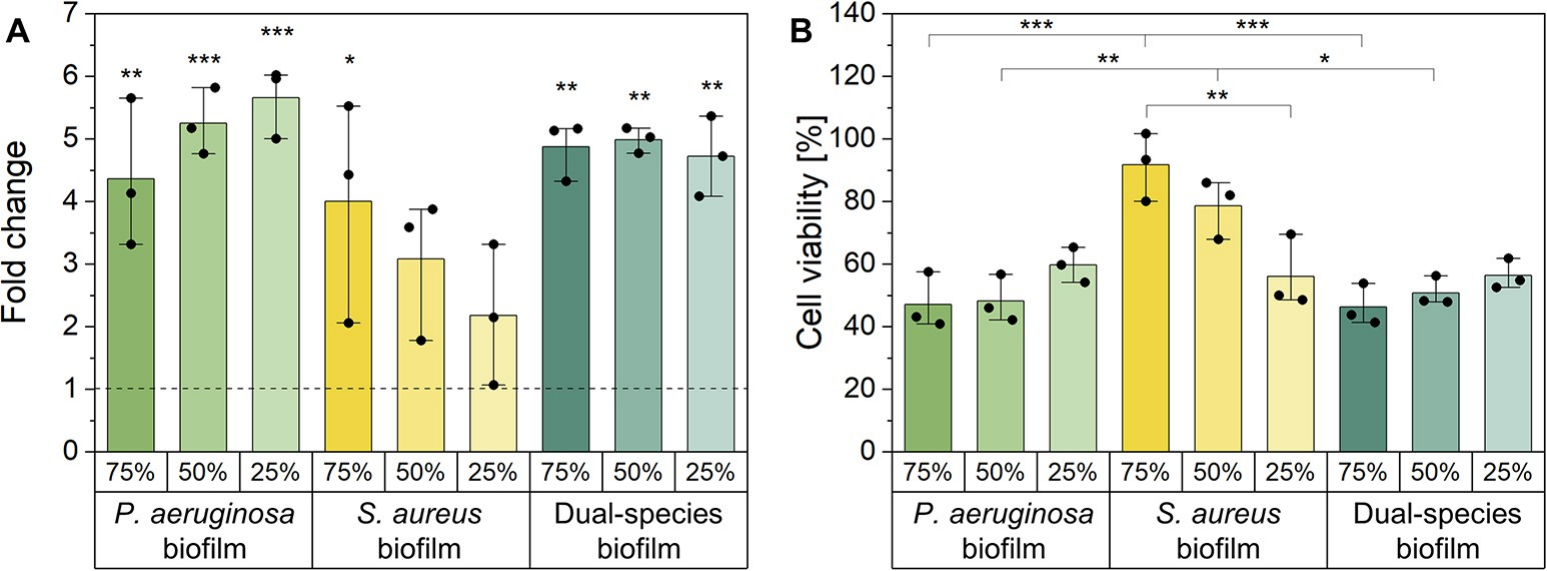
Analysis of the host cells’ pro-inflammatory response and viability following treatment with cell-free biofilm components. (A) The supernatant of scratch assays was used to evaluate the release of the cytokine IL-6 by an ELISA assay. Results are expressed as fold changes related to the untreated, wounded control. The dashed line indicates a fold change of 1. (B) Keratinocytes were treated with biofilm-conditioned media (BCM) from *P*. *aeruginosa* and *S*. *aureus* mono-species and dual-species biofilms in concentrations of 75%, 50%, and 25% to further determine the cell viability after BCM treatment by an MTT assay. The results were calculated in relation to the untreated control. *p < 0.05, **p < 0.01, and ***p < 0.001.

Since inflammation can delay wound healing and contribute to biofilm virulence, the immunostimulatory impact of the matrixome of the different biofilms was assessed. Quantification of the pro-inflammatory cytokine IL-6 in the scratch assay supernatant after 56 h revealed varying effects (Fig 7A). Cultivation with PA BCM resulted in a significantly, 4-6-fold, enhanced secretion of IL-6 compared to the control group of keratinoctytes cultured with unconditioned medium, reaching a maximum IL-6 concentration of 95.9 pg/mL. Notably, the fold changes increased as the concentration of the biofilm extract decreased. In general, the cultivation with SA BCM resulted in lower fold changes of IL-6 concentrations compared to PA BCM, a significant increase compared to the control was solely observed at a concentration of 75%, where the concentration of IL-6 in the supernatant peaked with 65.2 pg/mL. The incubation of keratinocytes with Dual BCM resulted in a significantly enhanced secretion of IL-6, up to approximately 5-fold changes compared to the control and a total concentration of 93.7 pg/mL. However, unlike PA BCM and SA BCM, the increased IL-6 secretion did not vary with the applied concentration.

The virulence of biofilm components can lead to a reduced cell viability of host cells, which may potentially impact wound healing in scratch assays. Therefore, the effect of BCM in concentrations from 25% to 75% on the viability of keratinocytes was evaluated with an MTT assay (Fig 7B). The treatment with PA BCM resulted in a pronounced reduction in cell viability for all tested concentrations. With decreasing concentration of PA BCM, a slight increase in viability of keratinocytes from about 47.0% (75% PA BCM) to 59.7% (25% PA BCM) was observed. Conversely, the viability of the cells treated with SA BCM correlated with the concentration applied. Treatment of cells with 75% SA BCM caused a slight reduction of their viability to 78.6%, leading to a significant difference compared to the PA BCM treatment. The reduction of the cell viability with 25% SA BCM to 56.0% was comparable to the treatment with 25% PA BCM. Keratinocytes subjected to Dual BCM showed a reduced viability equivalent to the viability observed after the treatment with PA BCM.

## Discussion

In this study, the development of biofilms and their functional properties in dependence of bacterial composition were investigated in the context of persistent wound infections. Even though the majority of wound associated biofilms in the clinics consists of more than one pathogen, little is known about the growth dynamics of dual species biofilms and their antibiotic susceptibility and virulence. Mono- as well as dual-species biofilms of *S*. *aureus* and *P*. *aeruginosa*, two of the most prevalent pathogens in wound infections were selected, which also cover both, Gram-positive and Gram-negative bacteria [5]. The respective biofilms were cultivated in a previously developed model system based on nanofibrous scaffolds fabricated via electrospinning [33]. This system precisely imitates the *in vivo* microenvironment of wound infections in terms of composition and structure, enhancing the relevance for the study of infectious biofilms in the clinical context [33].

### The dynamics of biofilm development regarding bacterial load and morphology

Initially, this study assessed the biofilm development based on quantification of adherent bacteria and evaluation of the biofilm morphology, in dependence of bacterial composition, including bacterial distribution, spatial organization, and matrix formation.

The development of mono-species biofilms of *P*. *aeruginosa* was characterized by a rapid colonization of the nanofibrous network, resulting in large bacterial aggregates and the production of EPS after 48 h. The increased accumulation of polysaccharides and production of pyocyanin further indicated an advanced development stage of *P*. *aeruginosa* biofilms [44–46]. In comparison, the development of biofilms of *S*. *aureus* was substantially delayed and key indicators of biofilm formation (bacterial aggregates, biomass density) were only found after 72 h.

Regarding the dynamics of biofilm development, there are hardly any comparative assessments with these two clinically relevant pathogens. Furthermore, the retrospective comparison of existing studies is hampered due to variations in selected strains, external growth conditions, and initial bacterial load [6, 47]. Nevertheless, it is acknowledged that *P*. *aeruginosa* exhibits a rapid capability for early biofilm formation [48, 49]. Further, e*x vivo* and *in vitro* studies focusing on a close imitation of native conditions in persistent wound infections consistently supported our findings, revealing an elevated number of *P*. *aeruginosa* adhering to wounded skin tissue and a higher bacterial load in *P*. *aeruginosa* mono-species biofilms compared to *S*. *aureus* [31, 50–52]. Previous studies relying on abiotic surfaces yielded contradictory results, highlighting the importance of adapting the *in vitro* growth conditions to the *in vivo* microenvironment [23].

Rapid biofilm formation was also observed in dual-species biofilms, with the formation of large aggregates of *P*. *aeruginosa* and *S*. *aureus*, and EPS visible after 48 h, indicating an advanced stage of biofilm development. At the first time point, the quantity of adherent *S*. *aureus* exceeded that in the mono-species biofilms, demonstrating cooperative interactions between the species. At later development stages, a progressive increase in the predominance of *P*. *aeruginosa* highlighted the shift towards more competitive interactions.

While both species co-exist in various tissue infections, their co-cultivation *in vitro* is still challenging due to an inherent dominance of *P*. *aeruginosa* over *S*. *aureus* [53, 54]. Different exoproducts secreted by *P*. *aeruginosa*, such as LasA protease or pyocyanin, have the potential to kill or inhibit the growth of *S*. *aureus* [23, 55]. While competitive interactions were reported to be predominant in planktonic phenotypes, chronic conditions, as found in persistent wounds, are known to facilitate cooperative mechanisms [23]. For instance, the presence of EPS of the biofilm matrix, as well as the downregulation of quorum sensing (QS) systems, can enable *S*. *aureus* survival alongside *P*. *aeruginosa* [56, 57]. Additionally, bacterial adhesion of *S*. *aureus* within the dual-species biofilms could be promoted by the formation of new binding sites resulting from early biofilm formation of *P*. *aeruginosa* [58]. The subsequent shift towards Gram-negative bacteria was analogous to reports of polymicrobial chronic wound infections in human [59]. Remarkably, the successful co-cultivation of both species over 72 h confirmed the similarity of growth conditions within the three-dimensional *in vitro* model system to native infected wound conditions.

Regarding the spatial organization of *P*. *aeruginosa* and *S*. *aureus* in co-cultivation, our findings revealed close proximity of both species. Earlier research demonstrated that during the initial stages of co-infection, this close contact plays a vital role in facilitating synergistic interspecies interaction [60]. Accordingly, other *in vitro* studies also revealed rather co-localization than self-segregation of both species during early biofilm formation [54, 61]. Samples of *in vivo* chronic wound infections indicated that both strains exist in separate communities [62]. However, it can be assumed that the process of self-segregation in biofilm-related wound infections requires significantly longer time periods.

### The antimicrobial tolerance with special focus on penetration in dependence on bacterial composition

One key functional property of bacterial biofilms studied in connection with bacterial composition was their distinct tolerance against antimicrobial treatments, which was assessed by susceptibility testing and penetration analysis of the antibiotic gentamicin. Confocal Raman microscopy was proven as valuable technique for label-free visualization of gentamicin in the biofilm, overcoming the limitations of labels which influence the penetration of the antibiotic substance. As non-invasive and label-free technique, confocal Raman microscopy has already been established for penetration analysis in other settings [63, 64]. Due to the fixation of samples prior to testing, the approach ensured the observation of physicochemical barrier properties of the biofilm matrix, independent of the pathogen’s susceptibility to the antibiotic.

A progressive increase of antibiotic tolerance with prolonged cultivation time was observed for mono-species biofilms of both, *S*. *aureus* and *P*. *aeruginosa*, despite differences in the biofilm’s development dynamics. The gentamicin penetration into the *P*. *aeruginosa* biofilms accordingly decreased with longer cultivation times. However, no significant dependence of the susceptibility and the penetration ability were observed for *S*. *aureus* mono-species biofilms.

It is generally recognized that the susceptibility to antimicrobial actives decreases during the transition from planktonic to biofilm phenotype bacteria [65]. The slightly reduced viability of *S*. *aureus* mono-species biofilms compared to *P*. *aeruginosa* biofilms was consistent with literature [65, 66]. It is important to emphasize that multiple mechanisms could contribute to the observed decrease in biofilm susceptibility during their development. The matrix composed of EPS was discussed as one factor in enhancing biofilm tolerance due to its role in restricting antibiotic penetration [20, 67]. This impediment partly arises from the gel-like viscosity which acts as a physical diffusion barrier [68]. Moreover, certain components of the matrix can selectively bind to and sequester antibiotics. Notably, prior research showed that aminoglycosides, such as gentamicin, interact with the polysaccharides of the *P*. *aeruginosa* biofilm matrix [69, 70]. In contrast, no such interaction was reported in the context of *S*. *aureus* biofilms [71]. Together with the overall looser morphology and reduced cell density of *S*. *aureus* biofilms this could explain that antibiotic penetration did not decrease with longer cultivation periods. Apart from the alteration of antibiotic penetration, factors like a decreased metabolic rate and the expression of protective factors, including multidrug efflux pumps or antibiotic-modifying enzymes, might determine the antibiotic tolerance of the biofilms [20, 72]. The broad spectrum of different defense mechanisms is one conceivable reason for the high viability of *S*. *aureus* biofilms despite sufficient antibiotic penetration.

For dual-species biofilms, a substantially greater capacity to withstand antibiotic treatments compared to mono-species biofilms was revealed. Based on the Raman analysis, a decreased penetration was identified as crucial contributor to antimicrobial tolerance. High tolerance of dual-species biofilms was also reported by previous *in vivo* and *in vitro* studies [54, 73], where the reduced penetration ability of antibiotics has been discussed as one of the most important tolerance mechanisms for dual‑species biofilms of *S*. *aureus* and *P*. *aeruginosa* compared to their mono-species counterparts [23]. However, prior to this study, no penetration analyses have been performed to confirm this assumption, to the best of our knowledge. Previously, it was demonstrated that the interaction of particular compounds of the biofilm matrix, including staphylococcal protein A (SpA) of *S*. *aureus* and Psl, an exopolysaccharide secreted by *P*. *aeruginosa*, enhanced the formation of large and dense biofilm aggregates, potentially explaining the observed reduction of antibiotic penetration [25, 74].

Interestingly, the sensitivity of *S*. *aureus* to the applied antibiotic rose substantially when cultivated within dual-species biofilms. Previous studies also reported competitive mechanisms of *P*. *aeruginosa*, decreasing the tolerance of *S*. *aureus* to antibiotic treatments [26, 27]. Still, it should be emphasized that no bactericidal effect occurred on *S*. *aureus* as viability remained clearly above 0.01%. This was also consistent with literature, as it was reported that these Gram-positive bacteria form highly tolerant small-colony variants (SCV) under stress conditions, including aminoglycoside treatments [75, 76].

### The impact of bacterial composition on biofilm virulence in the context of persistent wound infections

Biofilm virulence is affected by a variety of influence factors significantly contributing to a non-healing environment in the context of persistent wound infections. As recently reported, the matrixome represents one of the key factors determining biofilm virulence besides secreted exoproducts like toxins or enzymes [11]. To address this aspect, biofilm conditioned medium (BCM) served as cell-free surrogate for analysis of the host response with particular focus on wound healing, as well as cell viability and the host’s immune reaction.

*P*. *aeruginosa* (PA) BCM was found to significantly hinder *in vitro* wound healing for all concentrations applied, which was attributed to both, reduced cell viability as well as an enhanced pro-inflammatory response of human keratinocytes. The seemingly concentration-dependent decrease of the IL-6 secretion could be explained by the higher cell viability after treatment with lower concentrations of BCM. In contrast, the treatment with *S*. *aureus* (SA) BCM resulted in wound healing rates comparable to the control group, independent of the concentration applied, while the cell viability and IL-6 secretion decreased in correlation with the BCM concentration.

The results of PA BCM were consistent with previous studies [60, 77], whereas previous findings regarding SA BCM were highly variable. Consistent with the results of the present study, a proliferative effect of *S*. *aureus* exoproducts on human keratinocytes has been demonstrated in some studies [78, 79], while other studies reported a significantly reduced wound closure and a strong decrease in cell viability after SA BCM treatment [77, 80]. However, biofilms examined in the contradictory studies were cultivated on abiotic surfaces and the resulting variations in biofilm development could cause the diverging results. The cytotoxic effects of cell-free BCM derived from mono-species biofilms could be mediated by different exoproducts secreted by the respective bacteria, including proteases, exotoxins, and QS molecules [81, 82]. For *P*. *aeruginosa* biofilms, two of the exotoxins, ExoS and ExoT, were reported to additionally inhibit cell migration [81]. Notably, in the case of *S*. *aureus*, none of the exotoxins are known to influence the migration of keratinocytes into wounds [82]. This disparity could potentially explain the variations observed in the effects of the BCM on wound healing processes related to cell migration. Exoproducts and elements of the matrixome, such as pseudolysin and protease IV of *P*. *aeruginosa* as well as enterotoxin B and peptidoglycans of *S*. *aureus*, have been shown to induce the secretion of IL-6, either directly or via induction of apoptosis of host cells [83–87]. The relatively higher secretion of IL-6 following PA BCM treatment may be attributed to the fact that biofilm matrix components were found to be more effective at inducing IL-6 for *P*. *aeruginosa* compared to *S*. *aureus* biofilms [88]. Additionally, Hessle et al. suggested that Gram-negative bacteria generally exhibit a higher potency in inducing IL-6 secretion compared to Gram-positive bacteria [89]. However, in the present study, the storage of the BCM before application on the keratinocytes could also have an influence on the results. A previous study suggested that *S*. *aureus* activity is mediated by a protein, while *P*. *aeruginosa* activity is likely due to a small molecule [77]. When stored at -21°C, the BCM of *S*. *aureus* may be negatively affected.

Increased virulence of dual-species BCM over both mono-species BCMs was suggested by extensive cell detachment in scratch assays. However, this was not reflected by the overall cell viability as well as the pro-inflammatory response, which remained comparable to that of cells treated with PA BCM.

In general, the co-existence of *P*. *aeruginosa* and *S*. *aureus* in wound infections was shown to particularly delay wound healing and to result in elevated disease severity in both, *in vivo* and *in vitro* [55, 73, 90]. Several studies have demonstrated that the presence of *S*. *aureus* could stimulate the upregulation of crucial virulence factors of *P*. *aeruginosa* in dual-species biofilms, mediated by QS molecules or cell membrane components, leading to an increased virulence towards host cells [55, 91, 92]. The fact that the enhanced virulence did not result in reduced cell viability might potentially result from increased proliferation activity driven by *S*. *aureus*. Regarding the immune reaction, previous findings have been contradictory [93–96], highlighting the multifactorial nature of immune modulation by polymicrobial. The concentration-independent pro-inflammatory effect of dual-species BCM might further explain the pronounced virulence of multi-species biofilms observed *in vivo*, as even low concentrations of biofilm components are capable to induce high cytokine release, and thus maintaining a persistent inflammatory phase in wound infections.

The highest IL-6 concentrations observed in this study were comparable to the levels reported in clinical wound fluid samples, where a value of 125 pg/mL was presented as a cut-off to predict the presence of multi-species biofilms and the involvement of *P*. *aeruginosa* [97]. Thus, the amount of released IL-6 following the treatment with BCM was already extremely high. It can be assumed that the capacity of human keratinocytes to secrete IL-6 has been depleted. Differences of the pro-inflammatory potential of the BCMs beyond this point might not be detectable with this experiment.

## Conclusion

In this study, we provide a pioneering assessment of the role of bacterial composition in affecting biofilm development and its functional properties and impact on human skin cells under *in vivo*-relevant growth conditions. The comparative evaluation of *P*. *aeruginosa* and *S*. *aureus* biofilm models revealed species-dependent development dynamics and functional biofilm properties. Cultivation in a growth environment similar to the *in vivo* situation of wound infections further enabled the *in vitro* co-cultivation of both pathogens in dual-species biofilm models over an extended period of time. When compared to mono-species biofilm models, the observed effects of models including both species could not be attributed to either pathogen, nor was an additive relationship revealed. Instead, integrating both pathogens into dual-species biofilm models led to a combination of competitive and cooperative interactions, distinctively affecting the properties of each individual pathogen. The results indicate that it is not advisable to make predictions about antimicrobial resistance and biofilm virulence in multi-species biofilms based solely on the results from mono-species biofilms. Thus, the here presented approach highlights the importance of studying multi-species biofilms within context-relevant microenvironments and presents great potential for further applications. For instance, the incorporation of more pathogens, including anaerobic bacteria and fungi, even further extended cultivation periods, and the inclusion of host-pathogen interfaces can potentially enrich our understandings of polymicrobial communities. The here presented cultivation approach and comprehensive analysis procedures further provide a valuable tool set for translational research during the development of new treatment options of persistent polymicrobial infections.

## Materials and methods

### Materials

Nutrient broth was obtained from Becton Dickinson GmbH (Heidelberg, Germany). Dulbecco’s phosphate-buffered saline (PBS) was supplied by Biowest (Nuaillé, France). Ethanol and Formaldehyde methanol-free 30% were purchased from Carl Roth GmbH & Co. KG (Karlsruhe, Germany). Gentamicin sulfate was obtained from Fagron GmbH & Co. KG (Glinde, Germany). Cellulose acetate (CA, Mn 30.000), dimethyl sulfoxide (DMSO), fetal calf serum (FCS), gelatin (from porcine skin, 300 Bloom, type A) and methylthiazolyldiphenyltetrazoliumbromid (MTT) were obtained from Sigma-Aldrich (Steinheim, Germany). Dulbecco’s modified Eagle medium (DMEM), Human IL-6 Uncoated ELISA Set, McFarland equivalence turbidity standard 0.5, nutrient agar, cetrimide agar, mannitol salt agar, *Staphylococcus aureus* (*S*. *aureus*, ATCC 29213) and *Pseudomonas aeruginosa* (*P*. *aeruginosa*, ATCC 27853) were purchased from Thermo Fisher Scientific GmbH (Dreieich, Germany). Glacial acetic acid was sourced by VWR International GmbH (Darmstadt, Germany).

### Preparation of bacterial biofilms

Biofilms based on electrospun fiber scaffolds were prepared as previously described [29]. In brief, a homogenous solution was prepared by dissolving equal parts of cellulose acetate and gelatin in 90% acetic acid. Subsequently, this solution was electrospun into nanofibrous scaffolds following the process parameters stated in the aforementioned publication. Afterwards, round sections (12 mm diameter) of the electrospun scaffolds were inoculated with either *P*. *aeruginosa*, *S*. *aureus* or equal parts of both bacteria, resulting in a total bacterial count of approximately 1.5 * 10^6^ cells, adjusted using the McFarland equivalence turbidity standard 0.5. Biofilms were further cultivated at 37°C on modified nutrient agar plates, containing 20% FCS, and subjected to experiments after 24, 48 and 72 h of cultivation. Every 24 h, the modified nutrient agar plates were replaced with fresh plates.

### Scanning electron microscopy (SEM)

To visualize the biofilm morphology, samples were collected after 24, 48 and 72 h cultivation and fixed with 4% phosphate-buffered formaldehyde solution. The fixed samples were dehydrated using an ascending ethanol series and underwent complete ethanol removal by critical point drying (Leica EM CPD300 Automated Critical Point Dryer, Leica Mikrosysteme GmbH, Vienna, Austria). Subsequently, the biofilms were mounted on carbon tapes and sputter coated with gold / palladium for three minutes (SC7620, Quantum Design GmbH, Darmstadt, Germany). Micrographs were captured using a scanning electron microscope with an Everhart–Thornley secondary electron detector at a magnification of 2,000x and an acceleration voltage of 8 kV (EVO 10, Carl Zeiss Microscopy GmbH, Jena, Germany) at room temperature. This analysis was performed in triplicate.

### Quantification of colony forming units (CFUs)

The colony forming units (CFUs) of adherent *P*. *aeruginosa* and *S*. *aureus* in mono- and dual-species biofilms were determined at 24, 48 and 72 h cultivation. After two washing steps with PBS to remove planktonic bacteria, the biofilms were transferred to centrifuge tubes filled with zirconia beads and homogenized using a bead mill homogenizer (Bead Mill MAX, VWR International GmbH, Darmstadt, Germany). To obtain single cell suspensions, bacterial aggregates were subsequently disrupted by sonication. Serial 10-fold dilutions were prepared using sterile PBS and plated on nutrient agar plates. The plates were then incubated for 24 h at 37°C, after which all visible colonies were counted. To quantify the different bacterial species within dual-species biofilms, strain-specific agar plates were used for plate counting including ceramide agar for *P*. *aeruginosa* and mannitol salt agar for *S*. *aureus*. For CFU analysis, three independent experiments were performed.

### Confocal Raman microscopy

Using a confocal Raman microscope (alpha300R+, WITec GmbH, Ulm, Germany), biofilms were analyzed after a cultivation period of 24, 48 and 72 h. The diode laser with an excitation wavelength of 532 nm was adjusted to a laser power of 39 mW before the objective. For the analysis with a 63x water-dipping objective (NA 1.0), samples fixed with paraformaldehyde were placed on calcium fluoride slides and immersed in ddH2O. The spectra were recorded with a lateral resolution of 0.5 μm in each direction in combination with an integration time of 0.3 s per spectrum and a spectral resolution of 4 cm^-1^ in a range of 400–3700 cm^−1^. Prior to multivariate data analysis, the spectra underwent background subtraction using the WITec Project Plus software (WITec GmbH, Ulm, Germany). Subsequently, the spectra were subjected to z-score normalization, cosmic ray removal (moving median, threshold 10) and smoothing (Savitzky-Golay filter, polynomial order: 3, frame length: 9) using an in house written Matlab script (Version 2023a, Mathworks, USA). Afterwards, vertex component analysis was performed using the Matlab RamanLIGHT app [98], identifying endmembers which were used for the generation of false color images based on abundance maps. Raman scans were performed at three different areas of each biofilm.

### Antibiotic susceptibility testing

The antimicrobial tolerance of the different biofilms was investigated by determining the CFUs after treatment with the antibiotic gentamicin. Mono- and dual-species biofilms were cultivated for 24, 48 or 72 h and then transferred to a centrifuge tube containing 500 μl nutrient broth. To confirm the general antimicrobial effect of the treatment on planktonic bacteria, suspensions of *P*. *aeruginosa* and *S*. *aureus* were prepared by adjusting an overnight culture to 0.5 McFarland standard in nutrient broth. To maintain consistency with the biofilm samples, 500 μL of the bacterial suspensions were accordingly transferred to a centrifuge tube. The samples were treated with either 500 μl PBS or a gentamicin sulfate solution (20 μg / mL in PBS). After 24 h, the biofilm and planktonic samples were homogenized as described previously. Following sonication, bacterial suspensions were centrifuged, and the resulting pellets were resuspended in PBS. CFUs were quantified as stated above. The tolerance testing to the antibiotic treatment was performed in five independent experiments with one sample per condition.

### Penetration analysis

Paraformaldehyde-fixed biofilms were placed on calcium fluoride slides and incubated in a small Petri dish filled with 5 mL gentamicin solution (100 mg/mL, dissolved in ddH_2_O) for 1 h prior to the Raman analysis. For the measurement, a diode laser with an excitation wavelength of 785 nm was used, adjusted to a laser power of 50 mW before the objective. With a 63x water-dipping-objective, depth scans of the submerged biofilms were recorded with a resolution of 0.5 μm in z-direction and 1 μm in x-direction in combination with an integration time of 0.1 s per spectrum. For the evaluation, the spectra were preprocessed as described above, before hierarchical cluster analysis was performed to distinguish between the gentamicin solution above of the sample (gentamicin applied), the biofilms, and the calcium fluoride slide with an in-house written Matlab script (Version 2023a, Mathworks, USA). A schematic illustration of the three cluster is provided in S4 Fig. The area under the curve was calculated for the respective wavenumber range and the ratio between the gentamicin applied and the gentamicin in the sample was calculated. Three independent experiments were conducted with one sample per condition.

### Preparation of biofilm-conditioned medium

To obtain biofilm-conditioned medium (BCM) from 48 h biofilms, the different biofilms were cultivated, following the already described method. At the respective time point, the biofilms were homogenized and sonicated, as stated above. All bacterial cells were subsequently removed by centrifugation for 5 min at 4000 g and sterile filtration using a 0.2 μm filter. A total of four BCM samples were pooled for each condition and stored at -21°C until further use. Samples of three independent experiments were collected.

### Cell culture

The immortalized human keratinocytes (HaCaT) were kindly provided by Prof. N. Fusenig (German Cancer Research Center, Heidelberg, Germany) [99] and routinely cultured in high-glucose DMEM basal medium supplemented with 10% (v / v) FCS. The cells were maintained at 37°C in a humidified atmosphere containing 5% CO2 with medium change every 2 to 3 days. For further experiments, cells were seeded at a density of 10,000 cells per well in 48-well plates to reach a confluent monolayer within 5 days. Experiments were conducted with passage numbers ranging from 47 to 55.

### *In vitro* scratch wound healing assay

Prior to experimental wounding, the monolayers of HaCaT cells were carefully washed with sterile PBS. Next, scratches were created in the confluent monolayers using a 200 μL pipette tip. The samples were again washed with sterile PBS to remove any detached cells and debris from the scratches. Subsequently, they were incubated with BCM at different concentrations for 56 h and compared to cells treated with unconditioned medium as positive control. Light microscopic images were captured after 0, 8, 24, 36, 48 and 56 h with the bright field mode of a confocal laser scanning microscope (LSM 900, Carl Zeiss Microscopy GmbH, Jena, Germany) with the Axiocam 506 color camera (Carl Zeiss Microscopy GmbH, Jena, Germany) and a 10x objective (numeric aperture: 0.45, Plan-Apochromat, Carl Zeiss Microscopy GmbH, Jena, Germany). The wound size was analyzed using an inhouse written MATLAB script (Version R2023a, MathWorks, USA). The experiment was performed in triplicate using three different cell passages.

### Enzyme-linked immunosorbent assay

The supernatant of the *in vitro* scratch wound healing assays was collected 56 h after wounding and stored at -21°C up to 4 weeks to determine the concentration of interleukin-6 (IL-6). The assay was carried out using a Human IL-6 uncoated enzyme-linked immunosorbent assay (ELISA) set with a sensitivity of 2 pg / mL according to the manufacturer’s instructions. Using a microplate reader (Spark multimode microplate reader, Tecan, Maännerdorf, Switzerland), the absorbance was measured. Since the cell culture medium was supplemented with FCS, which inherently comprises cytokines, IL-6 concentration was also quantified in the medium and subtracted as background. The experiment was performed in triplicate for supernatants received from three independent scratch assays and results were expressed as fold changes compared to the positive control.

### Methylthiazolyldiphenyltetrazoliumbromid assay

The effect of the biofilm-conditioned media (BCM) on the metabolic activity of human epidermal cells (HaCaT) was assessed using a Methylthiazolyldiphenyltetrazoliumbromid (MTT) assay. After HaCaT cells reached a confluent monolayer, they were incubated with the BCM at different concentrations (25%, 50%, 75% and 100%) for 24 h at 37°C and 5% CO_2_. After the incubation period, the BCM containing media were removed and diluted MTT reagent was added, followed by another 4 h of incubation. The unreacted MTT reagent was then removed, and the resulting formazan crystals were dissolved in DMSO for 30 min. Untreated cells served as positive controls. The absorbance at 570 nm was measured using a microplate reader (Spark multimode microplate reader, Tecan, Maennerdorf, Switzerland) and the results were calculated as the percentage of metabolic activity according to Eq 1. Three biological replicates in three independent experiments were measured.


$$Metabolic\;activity\;[\%]=\frac{{absorbance}_{cells\;incubated\;with\;BCM\;}}{{absorbance}_{positive\;control}}\times 100\%$$ (Eq 1)

### Statistical analysis

All results were expressed as mean ± standard deviation (SD). The underlying raw data is listed in the S2 File. Comparison of different experimental groups were performed using one-way ANOVA, followed by Tukey’s post hoc test. For the statistical analysis of CFU quantification log_10_ CFU values were used. For CFU and MTT data, results were compared within similar bacterial compositions as well as similar cultivation periods or concentrations of BCM, respectively. For ELISA data, all results were compared to the control group. A p-value of p < 0.05 was considered statistically significant (p < 0.05 *, p < 0.01 **, p < 0.001 ***). The calculations were performed using Microsoft Office Excel 2016 and Origin Pro 2022.

## Supporting information

S1 Fig Scanning electron micrograph of nanofibrous model system for biofilm cultivation. Polymeric fiber networks of cellulose acetate and gelatin were fabricated via electrospinning and subsequently applied as three-dimensional scaffolds for biofilm formation. Scale bar: 10 μm.
(TIF)

S2 Fig Representative scanning electron micrograph to visualize extracellular polymeric substances (EPS) (A) of a mono-species biofilm of *P*. *aeruginosa* and (B) of a dual-species biofilm of *P*. *aeruginosa* and *S*. *aureus*. Arrows indicate strands of EPS. Scale bar: 1 μm.
(TIF)

S3 Fig Raman spectrum, false color image and peak assignment of the electrospun fiber network. (A) Raman spectrum of the electrospun fiber scaffold after 24 h on nutrient agar without bacteria and (B) the corresponding abundance map (scale bar = 5 μm). (C) Raman peaks of the electrospun fiber scaffold assigned to their vibrational mode and the corresponding polymers.
(TIF)

S4 Fig Schematic overview of the penetration analysis using Raman microscopy. (A) Experimental setup (B) False color image of a representative depth scan after hierarchical cluster analysis (C) Resulting mean spectra for each cluster. The area under the curve of the most prominent gentamicin peak at 976 cm^-1^ was calculated. Subsequently, the ratio between the gentamicin solution (gentamicin applied) and the gentamicin in the biofilm was calculated.
(TIF)

S1 File Antimicrobial effect of gentamicin on planktonic bacteria. To confirm the antimicrobial activity planktonic bacterial suspensions of *P*. *aeruginosa* as well as *S*. *aureus* were treated with the gentamicin solution. A reduction (log (CFUs/sample)) of above 3 indicates bactericidal effects.
(PDF)

S2 File Raw data. This file contains a data set that lists all the values used to calculate the means, standard deviations, and other measures reported in the study, as well as the values used to create the graphs.
(PDF)
