# Supplementary figures and images for: The interplay of Pseudomonas aeruginosa and Staphylococcus aureus in dual-species biofilms impacts development, antibiotic resistance and virulence of biofilms in in vitro wound infection models
Source: PLoS One. 2024 May 28;19(5):e0304491. doi: 10.1371/journal.pone.0304491 (PMC11132468; doi:10.1371/journal.pone.0304491)

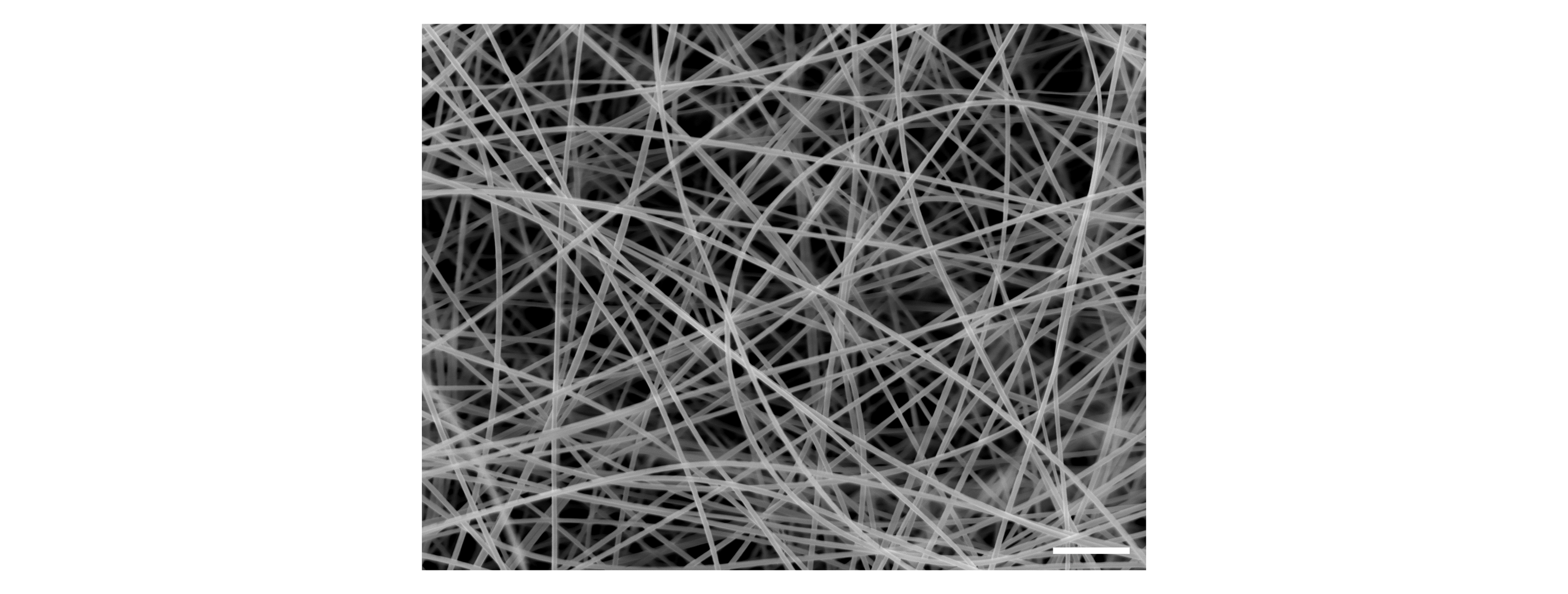

Supplement: S1 Fig — Polymeric fiber networks of cellulose acetate and gelatin were fabricated via electrospinning and subsequently applied as three-dimensional scaffolds for biofilm formation. Scale bar: 10 μm. (TIF) [file pone.0304491.s001.tif]

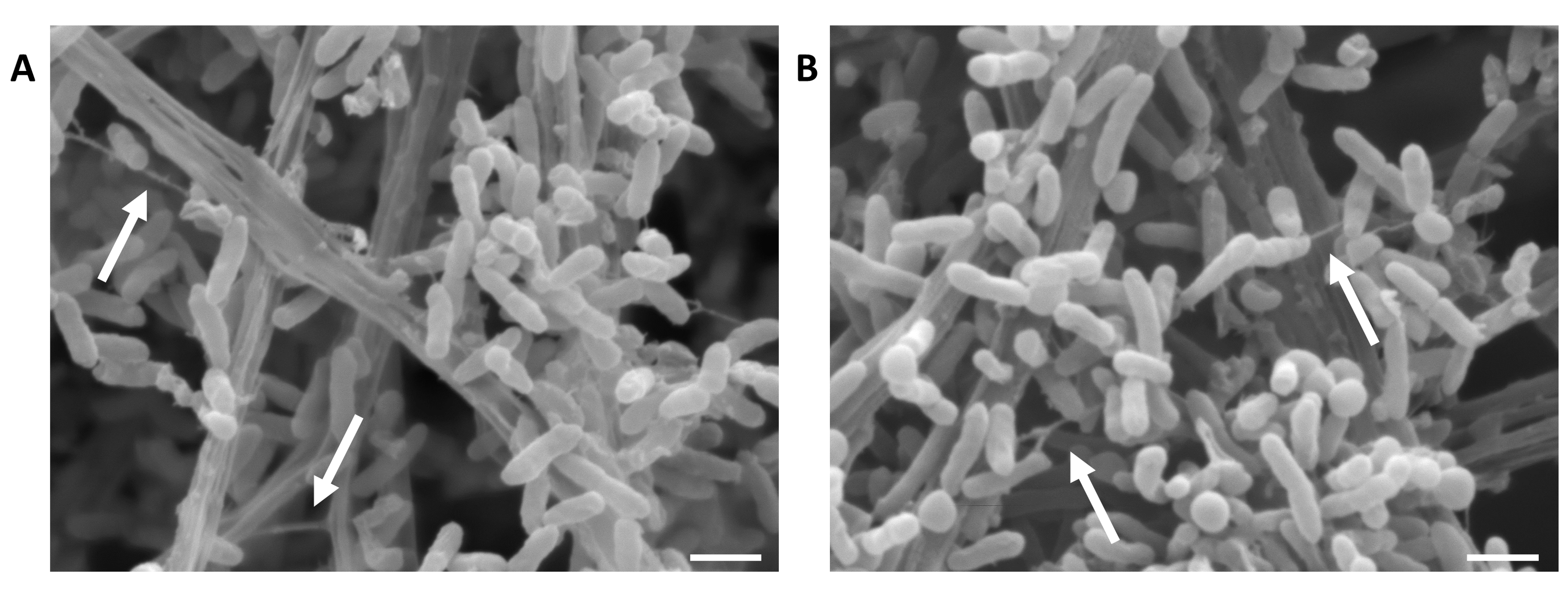

Supplement: S2 Fig — Representative scanning electron micrograph to visualize extracellular polymeric substances (EPS) (A) of a mono-species biofilm of P. aeruginosa and (B) of a dual-species biofilm of P. aeruginosa and S. aureus. Arrows indicate strands of EPS. Scale bar: 1 μm. (TIF) [file pone.0304491.s002.tif]

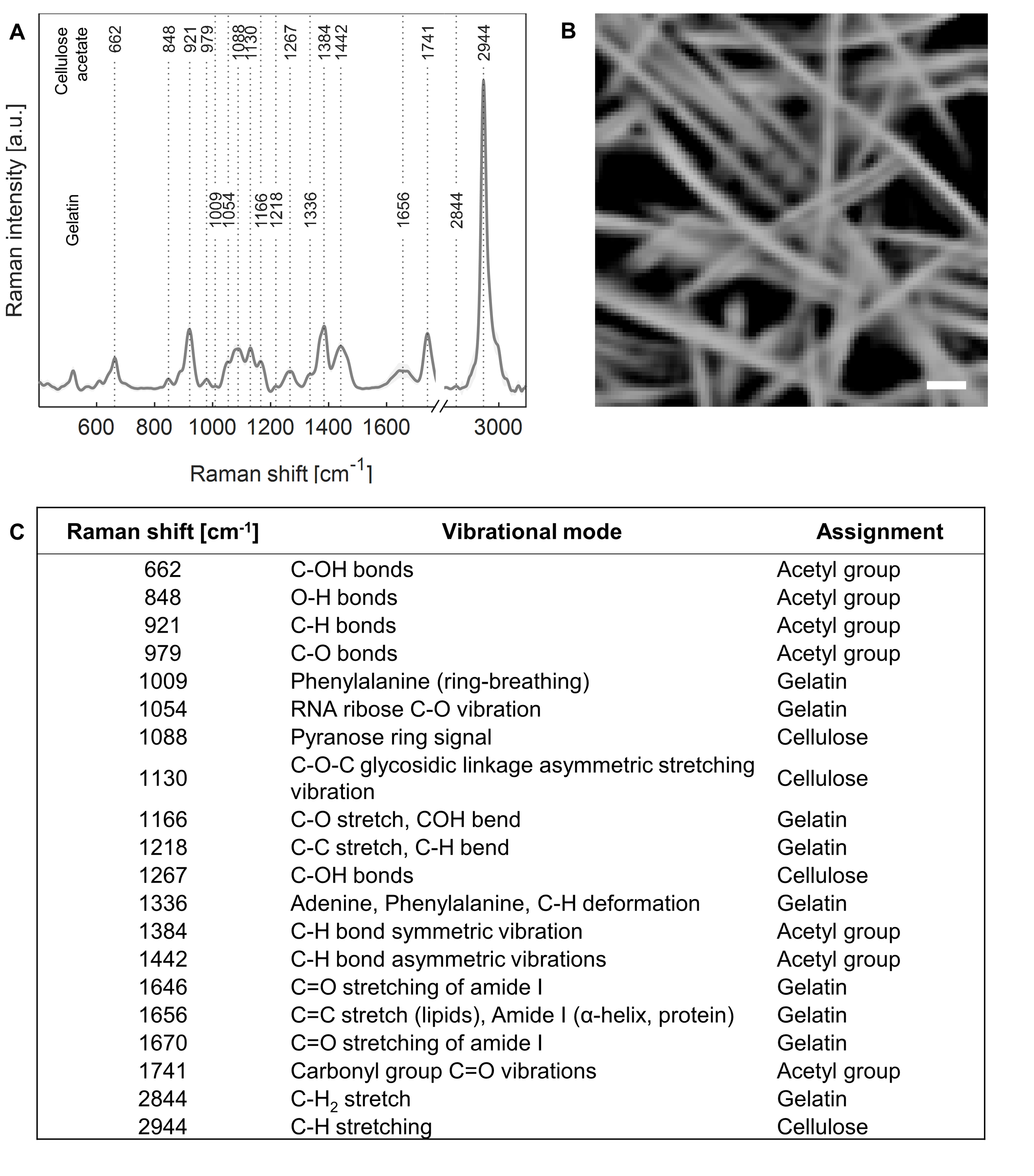

Supplement: S3 Fig — (A) Raman spectrum of the electrospun fiber scaffold after 24 h on nutrient agar without bacteria and (B) the corresponding abundance map (scale bar = 5 μm). (C) Raman peaks of the electrospun fiber scaffold assigned to their vibrational mode and the corresponding polymers. (TIF) [file pone.0304491.s003.tif]

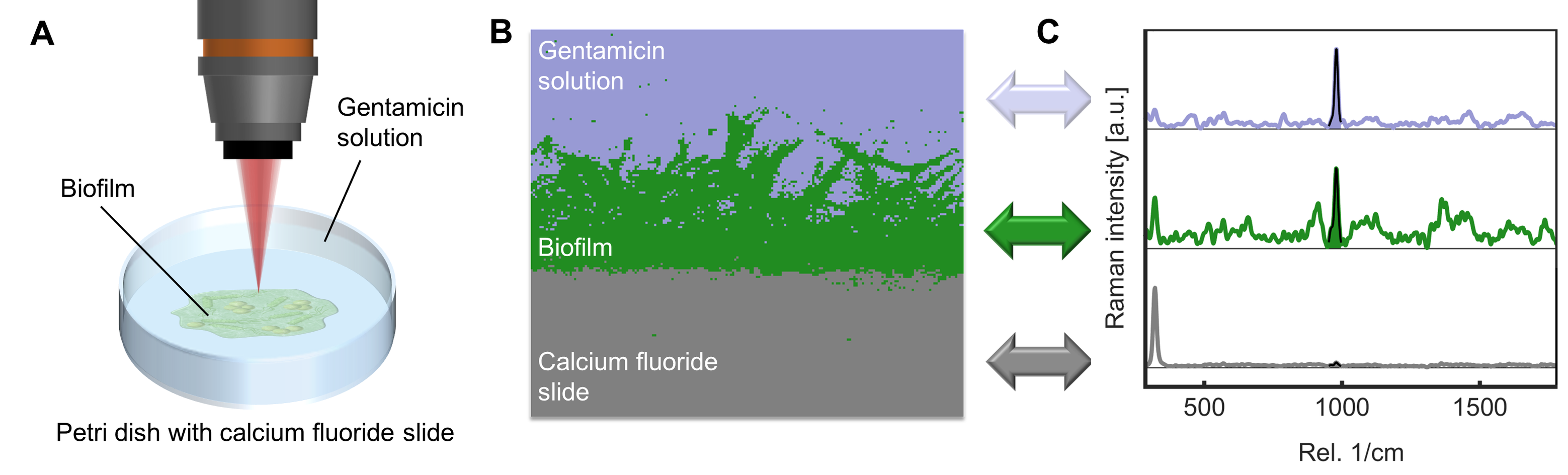

Supplement: S4 Fig — (A) Experimental setup (B) False color image of a representative depth scan after hierarchical cluster analysis (C) Resulting mean spectra for each cluster. The area under the curve of the most prominent gentamicin peak at 976 cm-1 was calculated. Subsequently, the ratio between the gentamicin solution (gentamicin applied) and the gentamicin in the biofilm was calculated. (TIF) [file pone.0304491.s004.tif]
