# Supplementary material for: The interplay of Pseudomonas aeruginosa and Staphylococcus aureus in dual-species biofilms impacts development, antibiotic resistance and virulence of biofilms in in vitro wound infection models
Source: PLoS One. 2024 May 28;19(5):e0304491. doi: 10.1371/journal.pone.0304491 (PMC11132468; doi:10.1371/journal.pone.0304491)
Supplement: S1 File — To confirm the antimicrobial activity planktonic bacterial suspensions of P. aeruginosa as well as S. aureus were treated with the gentamicin solution. A reduction (log (CFUs/sample)) of above 3 indicates bactericidal effects. (PDF) [file pone.0304491.s005.pdf]

Supporting Information: Antimicrobial effect of gentamicin on planktonic bacteria

P. aeruginosa (ATCC 27853) planktonic						
	log (CFU/sample)					
	Experiment 1	Experiment 2	Experiment 3	Experiment 4	**Mean**	**SD**
After treatment	2,70	3,40	2,48	3,73	**3,08**	**0,508749456**
Control	7,30	7,96	7,79	7,65	**7,68**	**0,280552959**
Reduction	4,60	4,56	5,32	3,92	**4,60**	**0,569864361**

S. aureus (ATCC 29213) planktonic						
	log (CFU/sample)					
	Experiment 1	Experiment 2	Experiment 3	Experiment 4	**Mean**	**SD**
After treatment	4,09	4,43	3,68	4,13	**4,08**	**0,267320245**
Control	7,35	7,48	7,48	7,39	**7,42**	**0,063284664**
Reduction	3,27	3,04	3,80	3,26	**3,34**	**0,319850852**
