# Supplementary material for: The interplay of Pseudomonas aeruginosa and Staphylococcus aureus in dual-species biofilms impacts development, antibiotic resistance and virulence of biofilms in in vitro wound infection models
Source: PLoS One. 2024 May 28;19(5):e0304491. doi: 10.1371/journal.pone.0304491 (PMC11132468; doi:10.1371/journal.pone.0304491)
Supplement: S2 File — This file contains a data set that lists all the values used to calculate the means, standard deviations, and other measures reported in the study, as well as the values used to create the graphs. (PDF) [file pone.0304491.s006.pdf]

Supporting Information: Raw data

Fig 2. Quantification of colony forming units of adherent biofilm bacteria.

P. aeruginosa biofilm				*S. aureus* biofilm			
Cultivation time	CFUs/sample			Cultivation time	CFUs/sample		
	Experiment 1	Experiment 2	Experiment 3		Experiment 1	Experiment 2	Experiment 3
24 h	2,32E+09	2,02E+09	2,59E+09	24 h	2,00E+07	6,90E+07	1,10E+08
48 h	2,22E+09	2,58E+09	3,21E+09	48 h	6,70E+07	5,80E+07	1,30E+08
72 h	2,41E+09	1,94E+09	3,28E+09	72 h	9,20E+08	4,88E+08	9,30E+08

Dual-species biofilm			
Cultivation time	CFUs/sample		
	Experiment 1	Experiment 2	Experiment 3
24 h	2,65E+09	3,10E+09	2,43E+09
48 h	2,66E+09	3,34E+09	3,15E+09
72 h	2,47E+09	2,87E+09	1,27E+09

Dual-species biofilm composition						
Cultivation time	CFUs/sample					
	P. aeruginosa			*S. aureus*		
	Experiment 1	Experiment 2	Experiment 3	Experiment 1	Experiment 2	Experiment 3
24 h	1,81E+09	1,59E+09	1,87E+09	8,40E+08	1,51E+09	5,60E+08
48 h	2,22E+09	2,22E+09	2,36E+09	4,40E+08	1,12E+09	7,90E+08
72 h	2,40E+09	2,80E+09	1,11E+09	7,20E+07	7,30E+07	1,60E+08

Fig 4. Susceptibility testing of the biofilms to the antibiotic gentamicin.

P. aeruginosa biofilm					
Cultivation time	Reductions (log10 CFUs/sample)				
	Experiment 1	Experiment 2	Experiment 3	Experiment 4	Experiment 5
24 h	0,77	0,53	0,61	0,46	1,03
48 h	0,49	0,46	0,15	0,19	0,51
72 h	0,07	0,45	0,12	0,17	0,26

S. aureus biofilm					
Cultivation time	Reductions (log10 CFUs/sample)				
	Experiment 1	Experiment 2	Experiment 3	Experiment 4	Experiment 5
24 h	0,76	0,88	0,47	0,60	1,47
48 h	0,91	0,39	0,36	0,61	0,24
72 h	0,16	0,52	0,09	0,30	0,38

Dual-species biofilm					
Cultivation time	Reductions (log10 CFUs/sample)				
	Experiment 1	Experiment 2	Experiment 3	Experiment 4	Experiment 5
24 h	0,55	0,63	0,18	0,61	0,72
48 h	-0,01	0,14	0,14	-0,08	0,22
72 h	-0,04	0,09	-0,03	0,03	0,03
**Dual-species biofilm composition after treatment**					
P. aeruginosa					
Cultivation time	Ratio [%]				
	Experiment 1	Experiment 2	Experiment 3	Experiment 4	Experiment 5
24 h	98,59%	96,98%	99,51%	99,46%	99,38%
48 h	93,10%	94,59%	89,65%	98,72%	98,23%
72 h	91,68%	99,84%	99,52%	99,78%	99,78%
S. aureus					
Cultivation time	Ratio [%]				
	Experiment 1	Experiment 2	Experiment 3	Experiment 4	Experiment 5
24 h	1,41%	2,39%	3,66%	1,34%	0,92%
48 h	4,50%	5,41%	10,66%	2,40%	1,50%
72 h	8,32%	0,21%	0,42%	0,20%	0,20%

Fig 5. Raman analysis of the penetration ability of gentamicin into the biofilms.

***P. aeruginosa* biofilm**				***S. aureus* biofilm**			
Cultivation time	Relative integrated Raman intensity [%]			Cultivation time	Relative integrated Raman intensity [%]		
	Experiment 1	Experiment 2	Experiment 3		Experiment 1	Experiment 2	Experiment 3
24 h	43,38%	34,54%	34,17%	24 h	29,33%	35,80%	22,71%
48 h	36,25%	26,21%	34,97%	48 h	36,32%	31,68%	45,16%
72 h	18,60%	24,76%	26,05%	72 h	32,90%	22,59%	32,36%

**Dual-species biofilm**			
Cultivation time	CFUs/sample		
	Experiment 1	Experiment 2	Experiment 3
24 h	31,90%	40,37%	36,85%
48 h	27,34%	28,07%	15,31%
72 h	13,37%	13,69%	10,37%

Fig 6. Scratch assays for evaluating the virulence of biofilm components regarding their effect on wound healing.

P. aeuruginosa **BCM**

	75% BCM			50% BCM			25% BCM		
	Wound closure [%]			Wound closure [%]			Wound closure [%]		
Cultivation time	Experiment 1	Experiment 2	Experiment 3	Experiment 1	Experiment 2	Experiment 3	Experiment 1	Experiment 2	Experiment 3
0h	0,00	0,00	0,00	0,00	0,00	0,00	0,00	0,00	0,00
8h	-5,72	5,25	-1,41	-0,76	12,64	2,83	3,34	15,24	3,44
24h	-6,67	11,68	-6,51	-0,93	14,86	4,03	5,67	20,84	13,84
32h	-8,59	13,87	-12,63	-1,43	15,88	-0,21	5,49	22,62	14,33
48h	-27,14	15,81	-22,95	-4,51	16,53	-6,40	3,41	26,03	10,19
56h	-45,79	15,54	-26,02	-7,47	17,58	-10,31	3,47	28,43	11,12

S. aureus **BCM**

	75% BCM			50% BCM			25% BCM		
	Wound closure [%]			Wound closure [%]			Wound closure [%]		
Cultivation time	Experiment 1	Experiment 2	Experiment 3	Experiment 1	Experiment 2	Experiment 3	Experiment 1	Experiment 2	Experiment 3
0h	0,00	0,00	0,00	0,00	0,00	0,00	0,00	0,00	0,00
8h	7,55	15,53	6,00	10,23	15,22	5,67	14,91	13,99	5,43
24h	26,42	29,50	43,96	38,34	34,93	45,89	48,16	36,49	51,84
32h	46,16	53,08	75,87	70,68	65,69	80,45	82,67	61,49	84,71
48h	91,76	95,90	97,70	99,80	99,63	98,64	99,20	97,27	98,04
56h	95,89	99,23	89,01	99,83	99,95	99,38	99,94	99,19	98,91

Dual-species **BCM**

	75% BCM			50% BCM			25% BCM		
	Wound closure [%]			Wound closure [%]			Wound closure [%]		
Cultivation time	Experiment 1	Experiment 2	Experiment 3	Experiment 1	Experiment 2	Experiment 3	Experiment 1	Experiment 2	Experiment 3
0h	0,00	0,00	0,00	0,00	0,00	0,00	0,00	0,00	0,00
8h	-26,26	9,38	-28,71	0,97	9,84	-0,04	4,43	10,85	0,26
24h	-24,92	10,94	-36,20	0,25	7,25	-3,62	6,77	9,26	8,60
32h	-25,55	8,30	-57,85	-1,21	6,31	-13,99	5,78	9,99	1,17
48h	-33,73	6,61	-80,77	-2,15	6,63	-24,62	5,04	10,97	-3,20
56h	-41,38	1,40	-101,76	-4,73	6,83	-33,93	4,21	12,68	-4,02

Control			
	Wound closure [%]		
Cultivation time	Experiment 1	Experiment 2	Experiment 3
0h	0,00	0,00	0,00
8h	9,99	17,59	3,32
24h	38,88	44,19	51,37
32h	57,84	65,89	76,97
48h	83,95	91,06	94,23
56h	89,83	94,50	94,84

Fig 7. Analysis of the host cells' pro-inflammatory response and viability following treatment with cell-free biofilm components.

(A) ELISA assay

P. aeruginosa **biofilm**				***S. aureus*** **biofilm**			
	Fold change				Fold change		
Concentration	Experiment 1	Experiment 2	Experiment 3	Concentration	Experiment 1	Experiment 2	Experiment 3
75%	5,65	3,31	4,13	75%	5,52	4,42	2,06
50%	5,82	5,17	4,76	50%	3,87	3,59	1,78
25%	6,01	5,96	5,00	25%	3,31	2,14	1,07

**Dual-species biofilm**			
	Fold change		
Concentration	Experiment 1	Experiment 2	Experiment 3
75%	5,16	4,32	5,13
50%	5,02	5,17	4,76
25%	4,72	5,36	4,08

(B) MTT assay

P. aeruginosa **biofilm**				***S. aureus*** **biofilm**			
	Cell viability [%]				Cell viability [%]		
Concentration	Experiment 1	Experiment 2	Experiment 3	Concentration	Experiment 1	Experiment 2	Experiment 3
75%	57,38	40,76	42,96	75%	101,57	93,30	80,05
50%	56,64	42,06	45,87	50%	81,86	85,96	67,79
25%	65,36	54,15	59,60	25%	48,49	69,50	49,90

**Dual-species biofilm**			
	Cell viability [%]		
Concentration	Experiment 1	Experiment 2	Experiment 3
75%	53,83	41,22	43,64
50%	56,17	47,85	48,10
25%	61,83	54,78	52,42
